# Outcomes among Patients with Mantle Cell Lymphoma Post-Covalent BTK Inhibitor Therapy in the United States: A Real-World Electronic Medical Records Study

**DOI:** 10.1155/2022/8262787

**Published:** 2022-12-28

**Authors:** Lisa M. Hess, Yongmei Chen, Paolo B. Abada, Heiko Konig, Richard A. Walgren

**Affiliations:** Eli Lilly and Company, Indianapolis, IN, USA

## Abstract

**Purpose:**

There remains a lack of consensus among experts regarding the optimal therapeutic approach for Mantle cell lymphoma (MCL) after failure of covalent Bruton Tyrosine Kinase inhibitor (cBTKi)-based therapy. This study was designed to examine patient characteristics, current treatment patterns, and clinical outcomes using a real-world database to evaluate how MCL is currently managed post-cBTKi therapy in the U.S.

**Methods:**

A large, deidentified U.S. electronic medical record (EMR) oncology database (ConcertAI) with data from January 2011 to July 2021 was utilized for this study. Eligible patients were adults with MCL who had received at least one cBTKi. Descriptive statistics were used to evaluate patient characteristics and treatment patterns. Time-to-event real-world outcomes of duration of therapy, time to next treatment discontinuation, and overall survival was evaluated using the Kaplan–Meier method.

**Results:**

A total of 946 patients met eligibility criteria. Of these, 739 (78.1%) discontinued cBTKi treatment before the end of the follow-up period, while the remaining 207 (21.9%) were still receiving cBTKi therapy at the end of the follow-up period. Among those who had discontinued the cBTKi, 352 (47.6%, 352/739) received at least one subsequent (post-cBTKi) treatment. The median duration of the immediate post-cBTKi therapy was 2.6 months (*n* = 352). Among the 739 patients who discontinued cBTKi treatment, the median time from cBTKi discontinuation to next treatment discontinuation or death was 3.9 months and the median overall survival was 10.3 months.

**Conclusions:**

This study demonstrates the poor outcomes experienced by patients after cBTKi therapy. There is an urgent need for safe and effective treatments for patients with recurrent or progressive MCL.

## 1. Introduction

Mantle cell lymphoma (MCL) is a rare and aggressive subtype of B-cell non-Hodgkin lymphoma (NHL). Approximately 75,769 patients were diagnosed with B-cell NHL in the U.S. in 2021, with 2.7% of these diagnosed with MCL (∼2,045 patients per year) [1]. MCL is more common in men (71% of all MCL diagnoses) and is most frequently diagnosed among patients aged 65 or older [2]. The incidence rate of MCL has remained relatively stable, only decreasing by 0.5% from 2008 to 2017 [1]. It is estimated that 61.9% of patients across all stages of disease live for 5 years or longer after a diagnosis of MCL [1]. Most patients present with advanced disease; as disease progresses, survival outcomes become increasingly poor [3]. After failure of a cBTKi, survival outcomes have been observed in the range of only 14 to 15 months [4].

Currently, there is a lack of consensus among experts regarding the optimal therapeutic approach for MCL and enrollment on a clinical trial is generally recommended, as none of the available treatments are considered curative. NCCN treatment guidelines for the initial treatment of MCL differ based on the stage of disease (stage I or stage II non-bulky disease versus stage II bulky or stage III-IV) [5]. Induction treatment for patients with stage II bulky disease through stage IV MCL has been primarily rituximab-based, but are further differentiated between aggressive versus less aggressive therapies. Aggressive therapies are utilized with consolidation treatment using high-dose chemotherapy followed by analogous stem cell rescue. Both aggressive and less aggressive therapies include single-agent rituximab maintenance therapy [5]. All patients will experience disease progression with advanced MCL. After progression, second-line treatment includes covalent Bruton Tyrosine Kinase inhibitor (cBTKi)-based therapy or additional rituximab-based therapy. Therapeutic options are limited for patients after having exhausted chemoimmunotherapy and cBTK inhibitors, with only a single therapy (brexucabtagene autoleucel) included within the 5.2021 NCCN treatment guidelines for MCL [5].

There is a lack of treatment options for patients with recurrent or progressive MCL following treatment with a cBTKi. Few data exist to quantify the outcomes of patients with MCL in the post-cBTKi treatment setting, where there are gaps in current care guidelines [5]. Prospective trial data in this setting include autologous T-cell (CAR T-cell) therapy, which has shown an objective response rate (ORR) of 93% and 61% of patients remained free of disease progression one year after treatment [6]. More recently, data from the phase 1/2 BRUIN trial demonstrated favorable efficacy associated with the use of pirtobrutinib (a novel noncovalent BTKi) among patients with MCL who had prior cBTKi treatment [7]. At the time of the interim analysis, 57% of all patients with MCL remained on treatment with no evidence of disease progression and the ORR rate was 52% among those with prior BTKi exposure [7].

Real-world data regarding outcomes of patients diagnosed with MCL are largely limited to treatment patterns and outcomes associated with first-line treatment or second-line cBTKi therapy [8–10]. Sparse data exist to evaluate the patient treatment pathway or outcomes after failure of cBTKi therapy, and optimal therapeutic options remain unknown [11]. Data regarding the outcomes of patients after cBTKi exposure have been limited to small retrospective studies, but consistently have shown poor outcomes, with median overall survival estimates ranging from 2.9 to 8.4 months [12–14].

This study was designed to quantify the treatment patterns and outcomes of patients after exposure to cBTKi therapies in routine clinical practice using a large contemporary database. Specifically, this study examined patient characteristics, current treatment patterns, and clinical outcomes using a real-world database of patients with MCL who have received cBTKi therapy in the U.S.

## 2. Methods

This retrospective observational study was designed to evaluate patient characteristics, treatment patterns, and outcomes (duration of therapy, time to treatment discontinuation, and overall survival) among patients with MCL who received a cBTKi.

### 2.1. Database

ConcertAI RWD360 is a deidentified clinical electronic medical record (EMR) oncology database that is built from multiple oncology health care clinics and oncologists that has been used for other research in hematology [15]. The database aggregates longitudinal, patient-level structured data from multiple oncology EMR systems using standard variable coding algorithms. The data used for this study include demographic information (e.g. age, sex), infused and oral therapies and dates each was received, as well as month and year of death. Data collected outside of the oncology EMR (e.g. imaging, stem cell therapy, hospitalizations, over-the-counter medications) are not included within the structured oncology EMR dataset. The RWD360 MCL database was developed by identifying all patients within the EMR systems with evidence of at least one International Classification of Disease (ICD) code reflective of a diagnosis of MCL (ICD-9: 200.4 or ICD-10: C83.1). RWD360 data are deidentified in accordance with standards of the HIPAA Privacy Rule through the suppression of patient health identifiers (PHI). PHI suppression was conducted in accordance with the expert determination method, and data used for this study are therefore not considered human subjects research per the US Code of Federal Regulations Section 45 [16]. Data were available from January 2011 through July 2021 at the time of analysis.

### 2.2. Cohort Selection

The first observation of an MCL diagnosis code was defined as the index date. Patients were included in the study cohort if the index date was between January 1, 2011 (start of the study period) through October 31, 2020. Data were available for follow-up through July 2021, which was considered the end of the study period. Additionally, patients were required to be 18 years of age or older at the index date and to have received systemic therapy with at least one drug that was included in the National Comprehensive Cancer Network (NCCN) treatment guidelines for MCL, version 5.2021 [5]. Lastly, the cohort was limited to patients who had received at least one cBTKi (ibrutinib, acalabrutinib, or zanubrutinib) on or after the index date.

### 2.3. Statistical Analyses

#### 2.3.1. Patient Characteristics and Treatment Sequences

Descriptive statistics were used to evaluate clinical and demographic characteristics at the start of the therapy for each subgroup and to describe the sequence of treatments received. Due to the large number of potential drug combinations, the following hierarchy was applied to simplify regimen reporting for the graphical summary of treatment sequences: cBTKi + BCL2i therapy (e.g., acalabrutinib + venetoclax, ibrutinib + venetoclax); cBTKi (e.g., acalabrutinib, ibrutinib) ± any other agents; BCL2i (e.g., venetoclax) ± any other agents; anti-CD20 therapy (e.g., rituximab) ± any other agents.

#### 2.3.2. Real-World Outcomes

Real-world outcomes included duration of therapy, time to next treatment discontinuation, and overall survival. Time-to-event analyses for real-world outcomes were conducted using the Kaplan–Meier method. Duration of therapy was defined as the time from the date of treatment initiation through the date of treatment discontinuation. Time to next treatment discontinuation was defined as the time from discontinuation of cBTKi therapy to the date of discontinuation of the next therapy or death (whichever occurred first). Since progression-free survival is not recorded in the ConcertAI RWD360 database, this analysis was used as a proxy to estimate the time to the next progression. This proxy estimate has been used in real-world studies of patients with solid tumors, but may underestimate the duration of time until progression in settings where discontinuation rates due to toxicity are high [17, 18]. Therefore, these data are simply reported as time to next treatment discontinuation or death and no direct inference is made with regards to progression. Patients were censored at the last observation if they were alive and had evidence of continuing to receive treatment within 60 days of the end of the database (end of the follow-up period), as it could not be determined with certainty if treatment had ended or may have continued but was not observed due to lack of additional follow-up in the database.

All analyses were conducted using SAS Enterprise 9.4 (Statistical Analysis Software, SAS Institute, Inc.) and treatment pattern images were created using Visual Studio Code (JavaScript, HTML, and CSS). No imputation was made for missing data; all missing variables were reported descriptively.

## 3. Results

### 3.1. Study Cohort

**Figure 1:**
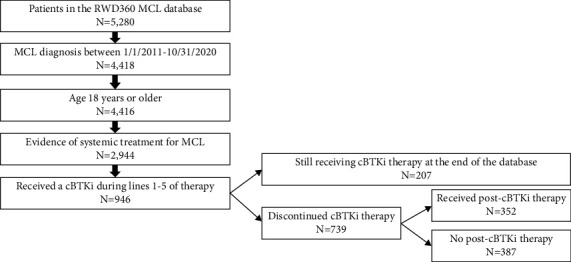
Flow chart of study cohort selection. Abbreviations: MCL = mantle cell lymphoma; cBTKi = covalent bruton tyrosine kinase inhibitor.

A total of 5,280 patient records were available in the ConcertAI RWD360 MCL database. A total of 946 patients met all eligibility criteria and were included in the final cohort for analysis. Of the 946 eligible patients, 739 (78.1%) discontinued the cBTKi before the end of the follow-up period, while the remaining 207 (21.9%) were still receiving cBTKi therapy at the end of the follow-up period. Among those who had discontinued the cBTKi, 352 (47.6%, 352/739) received at least one subsequent (post-cBTKi) treatment. A patient flow diagram that demonstrates the criteria applied to identify the final study cohort is included in Figure 1.

### 3.2. Patient Characteristics

The characteristics of the eligible study patient population are summarized in Table 1. The median age at the time of cBTKi initiation was 72 years and the cohort was predominantly male (75.1%). The majority of patients (67.3%) received care in community oncology practices. The characteristics of patients were similar regardless of the line of therapy in which the cBTKi was initiated (Supplementary Materials, Table S1).

### 3.3. Treatment Patterns

All patients by study design received at least one line of therapy. Of the 946 eligible patients, 670 (70.8%) had evidence of a second line of therapy, and 373 (39.4%) had evidence of a third line of therapy in the database. Most patients received cBTKi-based therapy in the first-line (*n* = 414, 43.8%) or second-line setting (*n* = 364, 38.5%). The most common first-line regimens included single-agent ibrutinib (*n* = 243, 25.7%) and rituximab plus bendamustine (*n* = 229, 24.2%). In the second-line setting, the most common regimens included single-agent ibrutinib (*n* = 200, 29.9%) and single-agent acalabrutinib (*n* = 88, 13.1%).

**Figure 2:**
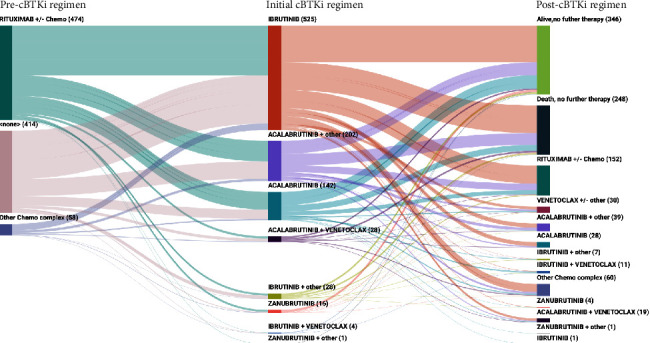
Treatment sequence prior to and following initial cBTKi. Abbreviations: cBTKi = covalent bruton tyrosine kinase inhibitor.

The sequence of treatments immediately prior to and immediately after the first cBTKi received is shown in Figure 2. Among the 352 patients who received at least one subsequent treatment following the cBTKi, the most common treatment regimens included rituximab (with or without other chemotherapy agents, *n* = 152, 43.2%). Thirty patients (8.5%) received BCL2i (venetoclax)-based therapy. Specific therapies such as bortezomib (*n* = 46, 13.1%) and lenalidomide (*n* = 44, 12.5%) were used infrequently, with or without other agents. A total of 146 (41.5%) received another cBTKi (with or without other agents) later in the course of therapy. A subset of patients received a cBTKi in more than one line of therapy: of all patients in this study, 29.5% received a cBTKi in both the first- and second-line setting and 25.3% received a cBTKi in both the second- and third-line setting.

Among the 946 eligible patients, 207 (21.9%) were still receiving a cBTKi at the time of data cutoff (end of the database) and 248 (26.2%) died before receiving post-cBTKi therapy. There were 346 (36.6%) that had no evidence of treatment after discontinuation of the initial cBTKi, and the reason for the lack of continued treatment could not be ascertained.

### 3.4. Real-World Outcomes

**Figure 3:**
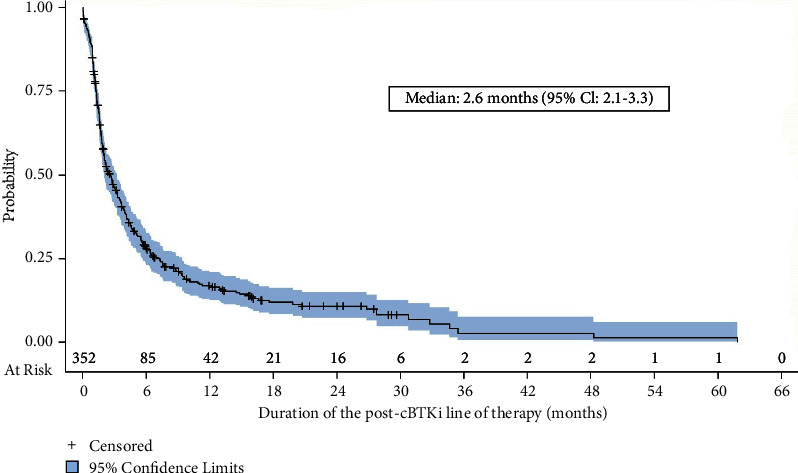
Duration of the immediate post-cBTKi line of therapy (*n* = 352). Abbreviations: cBTKi = covalent bruton tyrosine kinase inhibitor; CI = confidence interval.

**Figure 4:**
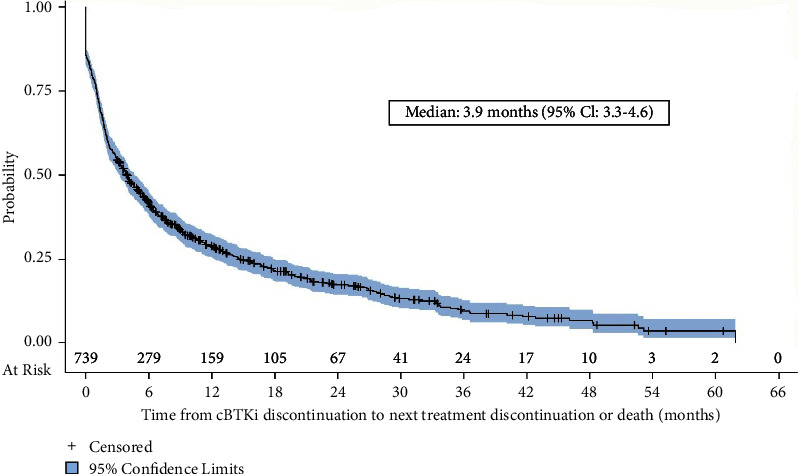
Time to next treatment discontinuation or death (*n* = 739). Abbreviations: cBTKi = covalent bruton tyrosine kinase inhibitor; CI = confidence interval.

For those patients who received additional treatment after discontinuation of the cBTKi, the median duration of the immediate post-cBTKi therapy was 2.6 months (95% confidence interval [CI]: 2.1–3.3, Figure 3). The duration of post-cBTKi therapy was short regardless of which line of therapy where the post-cBTKi therapy was received (no difference by line of therapy; *p* = 0.21, log-rank test, Supplementary Materials Figure S1). The median time to next treatment discontinuation (time from discontinuation of the cBTKi to the discontinuation of post-cBTKi therapy or death) was 3.9 months (95% CI: 3.3–4.6) (Figure 4). Time to next treatment discontinuation or death varied by the line of therapy in which the post-cBTKi treatment was initiated (median ranged from 6.6 months for those who received first-line cBTKi therapy to 1.7 months for those starting the cBTKi in the fourth line; *P* < 0.0001 (Supplementary Materials Figure S2).

**Figure 5:**
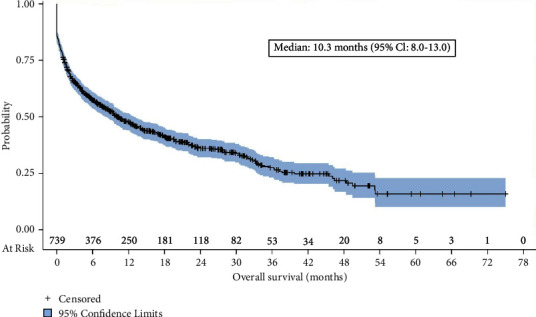
Overall survival from time of discontinuation of the cBTKi (*n* = 739). Abbreviations: cBTKi = covalent bruton tyrosine kinase inhibitor; CI = confidence interval.

Median overall survival was only 10.3 months (95% CI: 8.0–13.0) from the time of cBTKi discontinuation (Figure 5). Overall survival differed by the line of therapy in which the cBTKi was initiated (median survival ranged from 17.0 months for those who received first-line cBTKi therapy to 2.5 months for those starting the cBTKi in the fourth line; *P*=0.004 (Supplementary Materials, Figure S3).

## 4. Discussion

This study of a large real-world database demonstrates the poor outcomes experienced by patients with MCL after discontinuation of cBTKi therapy. This remains consistent with the overall pattern of declining outcomes observed in prior studies [3]. The prior work was limited to a single academic institution from 2000 to 2014, whereas this study adds to this earlier knowledge using a large database across multiple community and academic institutions with more recent data. This study used more contemporaneous data and further demonstrates that the outcomes after exposure to cBTKi therapy are particularly poor. For patients able to receive subsequent therapies, they have been used for a very short duration of time. Mortality outcomes after discontinuation of cBTKi therapy are particularly poor. This is not unexpected given the lack of recommended treatment options post-cBTKi therapy in the 2021 NCCN treatment guidelines, as nearly 40% of patients in this study did not receive additional therapy after cBTKi. In 2020, CAR T-cell therapy was approved for the care of patients with MCL. While current data do not allow for the evaluation of long-term outcomes of patients recently treated with these newer therapies, future research should evaluate the proportion of patients with MCL able to receive CAR T-cell therapy and the impact on patient overall survival. Due to the timeframe of the current database, this could not be explored.

The observation that nearly 40% did not receive additional treatment after cBTKi therapy could be in part due to the lack of treatment options available for the care of these patients. This is similar to other work in MCL more generally. Studies of real-world data in a similar time period have shown that 48.2% of patients receiving first-line therapy have evidence of second-line treatment, 47.6% of patients who receive a second line advance to receive a third-line treatment, and 44.1% of those who receive a third line go on to receive a fourth or later-line treatment [19]. However, other explanations could be relevant to explain the low rates of subsequent therapy but could not be evaluated. The current and other real-world studies may not have had sufficient follow-up to observe a subsequent treatment, or patients could have transferred to hospice or to another care setting where follow-up care could not be observed in this database. This is not fully evaluable in real-world datasets and could have led to an overestimation of patients who do not receive post-cBTKi therapy in the current study. While another real-world database study did not specifically evaluate post-cBTKi outcomes, survival from the start of third or later lines of therapy was a median of 18 months, [19] whereas in this study, survival was from the time of cBTKi discontinuation. While the studies evaluated different cohorts, both studies suggest that as disease progresses, outcomes worsen.

There was a high rate of cBTKi use in the first-line setting in this study, an observation which must be interpreted with caution. The specific line of therapy cannot be fully determined in the ConcertAI RWD360 dataset to the potential risk of missing data from prior health care that could have been received outside of the EMR recorded in this database. While data that may exist outside of the oncology EMR systems are unknown, the sequence of observed care is accurately recorded with dates of initiation and discontinuation. However, the high rates of cBTKi use early in the course of care could also be due in part be due to the study design, which required cBTKi exposure. This design element would have excluded patients who received chemoimmunotherapy or other anti-CD20-based treatments in the first-line setting and who had not yet progressed to second- or third-line cBTKi. Other possible explanations for the high use of first-line cBTKi therapy in this study could be the age of patients (median age of 72 years) and the treatment settings in which they received care (primarily community-based practices). Several international studies have suggested a preference for cBTKi therapy earlier in the care trajectory for the older patient, however, this has not yet been fully investigated [20–23]. Optimal care for the older patient with comorbidities or frailty remains unknown, but the likelihood for the need for a more tolerable regimen in the first-line setting cannot be dismissed. The majority of patients were cared for in community settings in this database, where oral therapies may be preferred as they are more easily and safely administered. Variation in care by practice setting should be studied in the future, as recent research focused on 12 large academic centers did not observe any cBTKi use in the first line among older patients with MCL, [24] whereas this largely community-based dataset showed utilization of these agents in what may be the first-line setting.

Limitations of this real-world dataset include the lack of data regarding the histology of MCL. Blastoid and pleomorphic MCL are associated with a particularly poor prognosis [25], and the patients in these data that exhibit this histology could not be identified. Future research is needed to further explore the outcomes post-cBTKi therapy among these particularly high-risk groups. As novel agents are developed, there is a need to evaluate the subpopulations that may benefit from different approaches to care in real-world settings.

As with any real-world dataset, limitations also include the nature of data collected in routine care. While these data provide insights regarding actual care patterns, the data are not collected for research purposes. This contributes to gaps in data, depending on the real-world data set used. For this database, performance status data were missing for nearly all patients, further limiting the ability to fully understand the patient populations included in this study. Data may have been captured at other healthcare facilities that were not included in the ConcertAI network. Data were also not available regarding reasons for treatment change. The lack of such variables (e.g. histology, performance status, comorbidities, biomarkers) for analysis limits the ability to identify clinically relevant subsets of patients, but may be possible in future studies that incorporate some level of chart review to extract additional variables for this type of study.

Despite the limitations of real-world data, this study shows the short duration of post-cBTKi therapy and poor overall survival experienced by patients after cBTKi therapy, and demonstrates the urgent need for safe and effective treatments for patients with MCL.

## Figures and Tables

**Table 1:** Patient characteristics.

Characteristic	Overall population (*N* = 946)	Patients who discontinued cBTKi therapy (*N* = 739)	Patients who received post-cBTKi therapy (*N* = 352)
Age, median years (range)	72.0 (33.1–88.5)^a^	72.7 (33.4–88.6)^b^	71.3 (36.2–88.6)^c^

*Sex, n (%)*			
Male	710 (75.1)	560 (75.8)	268 (76.1)
Female	236 (24.9)	179 (24.2)	84 (23.9)

*Race, n (%)*			
White	766 (81.0)	607 (82.1)	291 (82.7)
Black/African American	46 (4.9)	32 (4.3)	20 (5.7)
Asian	24 (2.5)	16 (2.2)	5 (1.4)
Other/unknown	110 (11.6)	84 (11.4)	36 (10.2)

*Geographic region, n (%)*			
Midwest	256 (27.1)	204 (27.6)	92 (26.1)
Northeast	98 (10.4)	71 (9.6)	34 (9.7)
South	371 (39.2)	285 (38.6)	151 (42.9)
West	196 (20.7)	161 (21.8)	67 (19.0)
Missing/unknown	25 (2.6)	18 (2.4)	8 (2.3)

*Practice setting, n (%)*			
Academic	298 (31.5)	239 (32.3)	117 (33.2)
Community	637 (67.3)	493 (66.7)	230 (65.3)
Missing/unknown	11 (1.2)	7 (0.9)	5 (1.4)

*Year of MCL diagnosis, n (%)*			
2011	46 (4.9)	41 (5.5)	24 (6.8)
2012	71 (7.5)	58 (7.8)	28 (8.0)
2013	98 (10.4)	88 (11.9)	33 (9.4)
2014	99 (10.5)	84 (11.4)	45 (12.8)
2015	132 (14.0)	107 (14.5)	52 (14.8)
2016	115 (12.2)	89 (12.0)	48 (13.6)
2017	133 (14.1)	106 (14.3)	43 (12.2)
2018	137 (14.5)	103 (13.9)	49 (13.9)
2019	77 (8.1)	46 (6.2)	23 (6.5)
2020	38 (4.0)	17 (2.3)	7 (2.0)

*Year of cBTKi discontinuation/last observed cBTKi (for those who did not discontinue)*			
2014	30 (3.2)	30 (4.1)	11 (3.1)
2015	63 (6.7)	62 (8.4)	31 (8.8)
2016	85 (9.0)	80 (10.8)	43 (12.2)
2017	111 (11.7)	98 (13.3)	45 (12.8)
2018	153 (16.2)	144 (19.5)	81 (23.0)
2019	167 (17.7)	155 (21.0)	70 (19.9)
2020	191 (20.2)	154 (20.8)	62 (17.6)
2021	146 (15.4)	16 (2.2)	9 (2.6)
Time from initial diagnosis, median months (range)	14.4 (0–116.4)^d^	24.0 (0.2–114.4)^e^	25.2 (1.2–114.8)^f^

Abbreviations: cBTKi = covalent bruton tyrosine kinase inhibitor; MCL = mantle cell lymphoma. ^a^age at start of cBTKi therapy, ^b^age at cBTKi discontinuation, ^c^age at the start of post-cBTKi therapy, ^d^time from initial diagnosis of MCL to start of cBTKi therapy, ^e^time from initial diagnosis of MCL to cBTKi discontinuation, ^f^time from initial diagnosis of MCL to start of post-cBTKi therapy.

## Data Availability

The data that support the findings of this study are obtained by license from ConcertAI, Inc. These deidentified data may be made available upon reasonable request from ConcertAI, and are subject to a license agreement.

## Supplementary Materials

Supplementary Materials The supplementary content includes descriptive and outcome summaries (duration of therapy, time to next treatment discontinuation or death, overall survival) by line of therapy in which the cBTKi was received.

## References

[1] Howlader N., Noone A. M., Krapcho M. (2020). *SEER Cancer Statistics Review, 1975-2017*.

[2] Cheah C. Y., Seymour J. F., Wang M. L. (2016). Mantle cell lymphoma. *Journal of Clinical Oncology*.

[3] Kumar A., Sha F., Toure A. (2019). Patterns of survival in patients with recurrent mantle cell lymphoma in the modern era: progressive shortening in response duration and survival after each relapse. *Blood Cancer Journal*.

[4] Hess G., Dreyling M., Oberic L. (2021). *KTE-X19 versus Standard of Care for Relapsed/refractory Mantle Cell Lymphoma Previoulsy Treated with Bruton Tyrosine Kinase Inhibitors: Real-World Evidence Form Europe*.

[5] Nccn N. C. CN. (2021). Clinical Practice Guidelines in Oncology: B-Cell Lymphomas. *Vox*.

[6] Wang M., Munoz J., Goy A. (2020). KTE-X19 CAR T-cell therapy in relapsed or refractory mantle-cell lymphoma. *New England Journal of Medicine*.

[7] Mato A. R., Shah N. N., Jurczak W. (2021). Pirtobrutinib in relapsed or refractory B-cell malignancies (BRUIN): a phase 1/2 study. *The Lancet*.

[8] Shah B. D., Yang K., Liu S. (2021). Real-world Bruton tyrosine Kinase inhibitor treatment patterns, compliance, costs, and hospitalizations in patients with mantle cell lymphoma in the United States. *Blood*.

[9] Goyal R. K., Jain P., Nagar S. P. (2021). Real-world evidence on survival, adverse events, and health care burden in Medicare patients with mantle cell lymphoma. *Leukemia and Lymphoma*.

[10] Kabadi S. M., Byfield S. D., Le L., Olufade T. (2021). Adverse events and economic burden among patients receiving systemic treatment for mantle cell lymphoma: a real-world retrospective cohort study. *Anticancer Research*.

[11] Eyre T. A., Cheah C. Y., Wang M. L. (2022). Therapeutic options for relapsed/refractory mantle cell lymphoma. *Blood*.

[12] Martin P., Maddocks K., Leonard J. P. (2016). Postibrutinib outcomes in patients with mantle cell lymphoma. *Blood*.

[13] Cheah C., Chihara D., Romaguera J. E. (2015). Patients with mantle cell lymphoma failing ibrutinib are unlikely to respond to salvage chemotherapy and have poor outcomes. *Annals of Oncology*.

[14] Rai S., Tanizawa Y., Cai Z. (2021). MCL-041: outcomes for recurrent mantle cell lymphoma post-BTK inhibitor therapy in Japan: an administrative database study. *Clinical Lymphoma, Myeloma & Leukemia*.

[15] Mato A. R., Hess L. M., Chen Y. (2022). Outcomes for Patients with Chronic Lymphocytic Leukemia (CLL) Previously Treated with Both a Covalent BTK and BCL2 Inhibitor in the United States: A Real-World Database Study. *Clinical Lymphoma Myeloma and Leukemia*.

[16] Hhs (2020). Human subject Regulations decision charts: 2018 requirements. https://www.hhs.gov/ohrp/regulations-and-policy/decision-charts-2018/index.html#c1.

[17] Blumenthal G., Gong Y., Kehl K. (2019). Analysis of time-to-treatment discontinuation of targeted therapy, immunotherapy, and chemotherapy in clinical trials of patients with non-small-cell lung cancer. *Annals of Oncology*.

[18] Boegemann M., Khaksar S., Bera G. (2019). Abiraterone acetate plus prednisone for the Management of Metastatic Castration-Resistant Prostate Cancer (mCRPC) without prior use of chemotherapy: report from a large, international, real-world retrospective cohort study. *BMC Cancer*.

[19] Narkhede M., Goyal G., Shea L., Mehta A., Giri S. (2022). Evaluating real-world treatment patterns and outcomes of mantle cell lymphoma. *Blood Advances*.

[20] McCulloch R., Lewis D., Crosbie N. (2021). Ibrutinib for mantle cell lymphoma at first relapse: a United Kingdom real‐world analysis of outcomes in 211 patients. *British Journal of Haematology*.

[21] Yang X., Khoo L. P., Chang E. W. Y. (2021). Treatment patterns and outcomes of older patients with mantle cell lymphoma in an Asian population. *BMC Cancer*.

[22] Rampotas A., Wilson M. R., Lomas O. (2021). Treatment patterns and outcomes of unfit and elderly patients with Mantle cell lymphoma unfit for standard immunochemotherapy: a UK and Ireland analysis. *British Journal of Haematology*.

[23] Ruan J. (2020). Approach to the initial treatment of older patients with mantle cell lymphoma. *Hematology-Oncology Clinics of North America*.

[24] Karmali R., Switchenko J. M., Goyal S. (2021). Multi‐center analysis of practice patterns and outcomes of younger and older patients with mantle cell lymphoma in the rituximab era. *American Journal of Hematology*.

[25] Dreyling M., Klapper W., Rule S. (2018). Blastoid and pleomorphic mantle cell lymphoma: still a diagnostic and therapeutic challenge!. *Blood*.

