# Supplementary material for: Outcomes among Patients with Mantle Cell Lymphoma Post-Covalent BTK Inhibitor Therapy in the United States: A Real-World Electronic Medical Records Study
Source: Adv Hematol. 2022 Dec 28;2022:8262787. doi: 10.1155/2022/8262787 (PMC9812614; doi:10.1155/2022/8262787)
Supplement: Supplementary Materials — The supplementary content includes descriptive and outcome summaries (duration of therapy, time to next treatment discontinuation or death, overall survival) by line of therapy in which the cBTKi was received. [file 8262787.f1.zip › Supplementary file description.docx]

Supplementary file information:

The supplementary content includes descriptive and outcome summaries (duration of therapy, time to next treatment discontinuation or death, overall survival) by line of therapy in which the cBTKi was received.
