# Supplementary material for: Outcomes among Patients with Mantle Cell Lymphoma Post-Covalent BTK Inhibitor Therapy in the United States: A Real-World Electronic Medical Records Study
Source: Adv Hematol. 2022 Dec 28;2022:8262787. doi: 10.1155/2022/8262787 (PMC9812614; doi:10.1155/2022/8262787)
Supplement: Supplementary Materials — The supplementary content includes descriptive and outcome summaries (duration of therapy, time to next treatment discontinuation or death, overall survival) by line of therapy in which the cBTKi was received. [file 8262787.f1.zip › Supplementary material 11-18-22.docx]

**Supplementary Materials**

**Table S1. Patient characteristics by line of therapy in which the cBTKi was initiated**

| **Characteristic** | **First line**  **(n=414)** | **Second line**  **(n=364)** | **Third line (n=115)** | **Fourth line (n=38)** | **Fifth line**  **(n=15)** |
| --- | --- | --- | --- | --- | --- |
| Age, median years (range) at start of cBTKi | 72.1 (36.0-88.1) | 73.2 (44.9-88.5) | 70.4 (42.3-88.0) | 64.4 (33.1-86.3) | 65.2 (39.2-84.1) |
| Sex, n (%) |  |  |  |  |  |
| Male | 316 (76.3) | 267 (73.4) | 85 (73.9) | 30 (78.9) | 12 (80.0) |
| Female | 98 (23.7) | 97 (26.6) | 30 (26.1) | 8 (21.1) | 3 (20.0) |
| Race, n (%) |  |  |  |  |  |
| White | 343 (82.9) | 288 (79.1) | 95 (82.6) | 28 (73.7) | 12 (80.0) |
| Black/African American | 15 (3.6) | 18 (4.9) | 8 (7.0) | 4 (10.5) | 1 (6.7) |
| Asian | 14 (3.4) | 5 (1.4) | 4 (3.5) | 1 (2.6) | 0 (0.0) |
| Other/unknown | 42 (10.1) | 53 (14.6) | 8 (7.0) | 5 (13.2) | 2 (13.3) |
| Geographic region, n (%) |  |  |  |  |  |
| Midwest | 116 (28.0) | 96 (26.4) | 26 (22.6) | 13 (34.2) | 5 (33.3) |
| Northeast | 29 (7.0) | 44 (12.1) | 18 (15.7) | 5 (13.2) | 2 (13.3) |
| South | 160 (38.6) | 152 (41.8) | 43 (37.4) | 10 (26.3) | 6 (40.0) |
| West | 97 (23.4) | 64 (17.6) | 24 (20.9) | 9 (23.7) | 2 (13.3) |
| Missing/unknown | 12 (2.9) | 8 (2.2) | 4 (3.5) | 1 (2.6) | 0 (0.0) |
| Practice setting, n (%) |  |  |  |  |  |
| Academic | 146 (35.3) | 86 (23.6) | 43 (37.4) | 19 (50.0) | 4 (26.7) |
| Community | 261 (63.0) | 274 (75.3) | 72 (62.6) | 19 (50.0) | 11 (73.3) |
| Missing/unknown | 7 (1.7) | 4 (1.1) | 5 (4.3) | 0 (0.0) | 0 (0.0) |
| Year of MCL diagnosis, n (%) |  |  |  |  |  |
| 2011 | 22 (5.3) | 10 (2.7) | 7 (6.1) | 3 (7.9) | 4 (26.7) |
| 2012 | 21 (5.1) | 24 (6.6) | 18 (15.7) | 5 (13.2) | 3 (20.0) |
| 2013 | 38 (9.2) | 34 (9.3) | 16 (13.9) | 6 (15.8) | 4 (26.7) |
| 2014 | 40 (9.7) | 42 (11.5) | 15 (13.0) | 1 (2.6) | 1 (6.7) |
| 2015 | 53 (12.8) | 56 (15.4) | 19 (16.5) | 4 (10.5) | 0 (0.0) |
| 2016 | 52 (12.6) | 49 (13.5) | 9 (7.8) | 4 (10.5) | 1 (6.7) |
| 2017 | 61 (14.7) | 53 (14.6) | 9 (7.8) | 8 (21.1) | 2 (13.3) |
| 2018 | 68 (16.4) | 54 (14.8) | 10 (8.7) | 5 (13.2) | 0 (0.0) |
| 2019 | 37 (8.9) | 31 (8.5) | 7 (6.1) | 2 (5.3) | 0 (0.0) |
| 2020 | 22 (5.3) | 11 (3.0) | 5 (4.3) | 0 (0.0) | 0 (0.0) |
| Time from initial diagnosis to start of cBTKi, median months (range) | 6.7 (0-105.1) | 15.5 (1.5-110.2) | 29.6 (3.3-116.4) | 26.7 (7.1-99.1) | 37.6 (18.4-105.8) |

Abbreviations: cBTKi= covalent Bruton Tyrosine Kinase inhibitor

**Figure S1. Duration of the immediate post-cBTKi line of therapy, by line in which the post-cBTKi regimen was initiated**

**
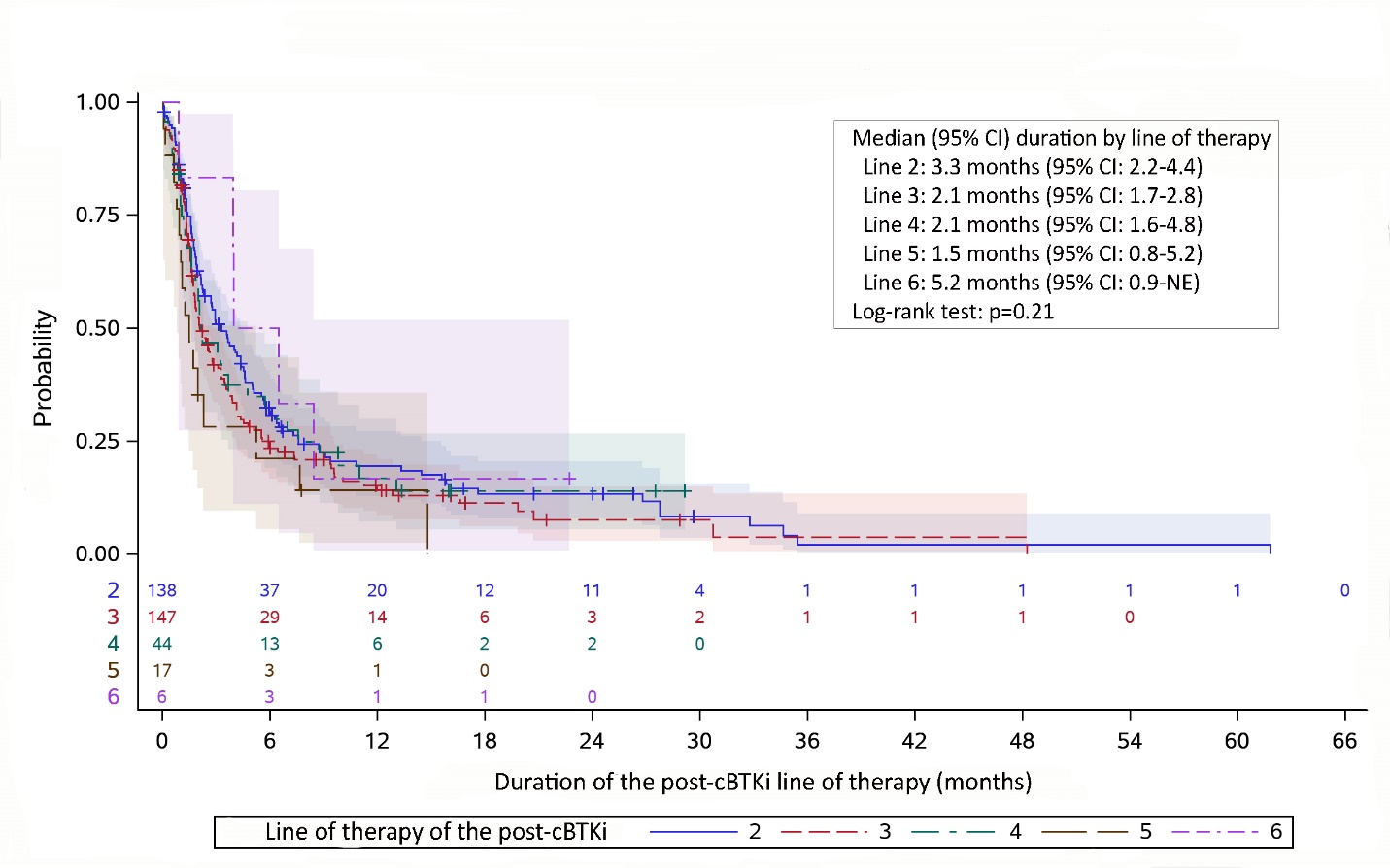
**

Abbreviations: cBTKi= covalent Bruton Tyrosine Kinase inhibitor; CI=confidence interval; NE=not evaluable

**Figure S2. Time to next treatment discontinuation or death, by line of therapy in which the cBTKi regimen was initiated**

**
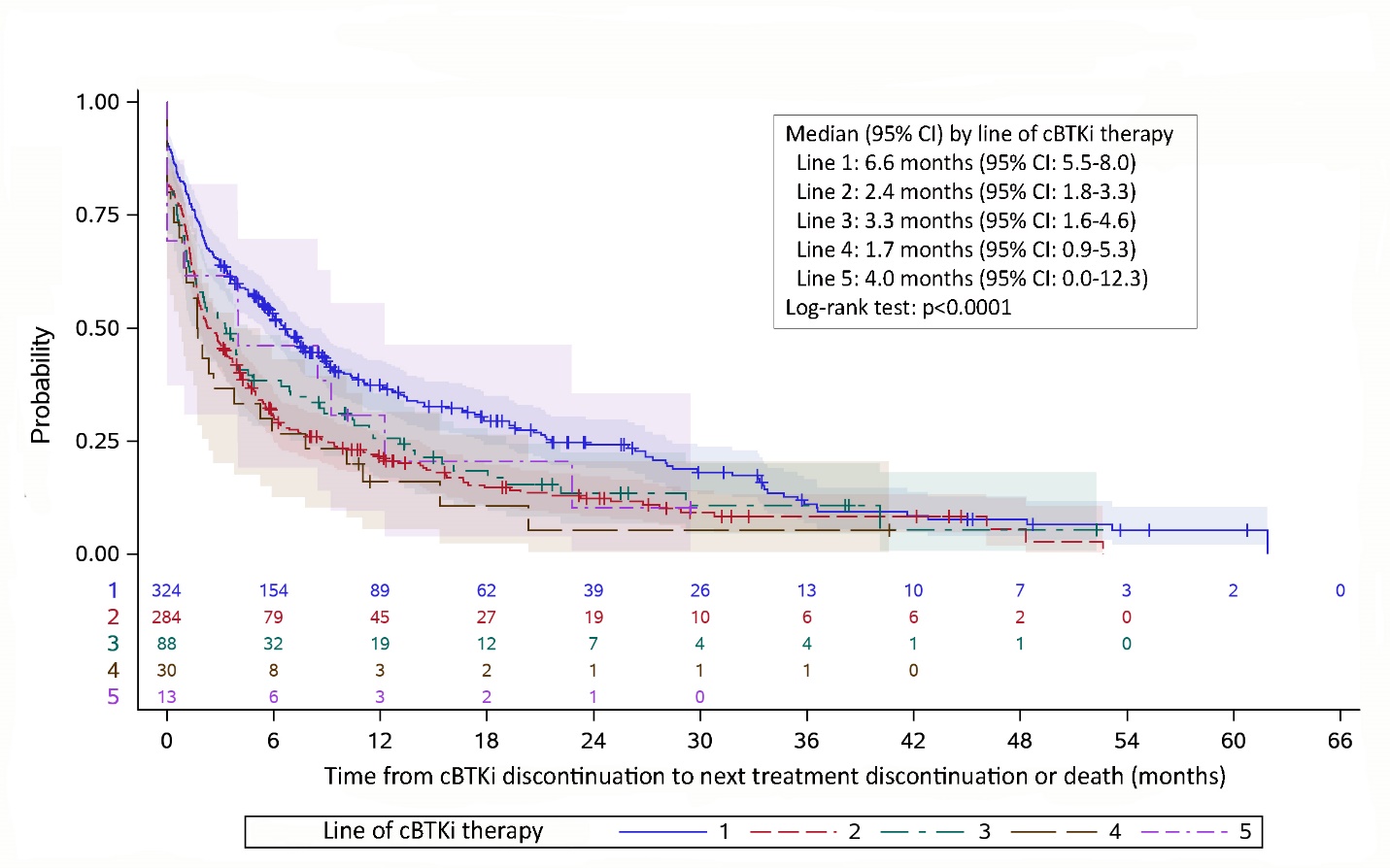
**

Abbreviations: cBTKi= covalent Bruton Tyrosine Kinase inhibitor; CI=confidence interval

**Figure S3. Overall survival, by line of therapy in which the cBTKi regimen was initiated**

**
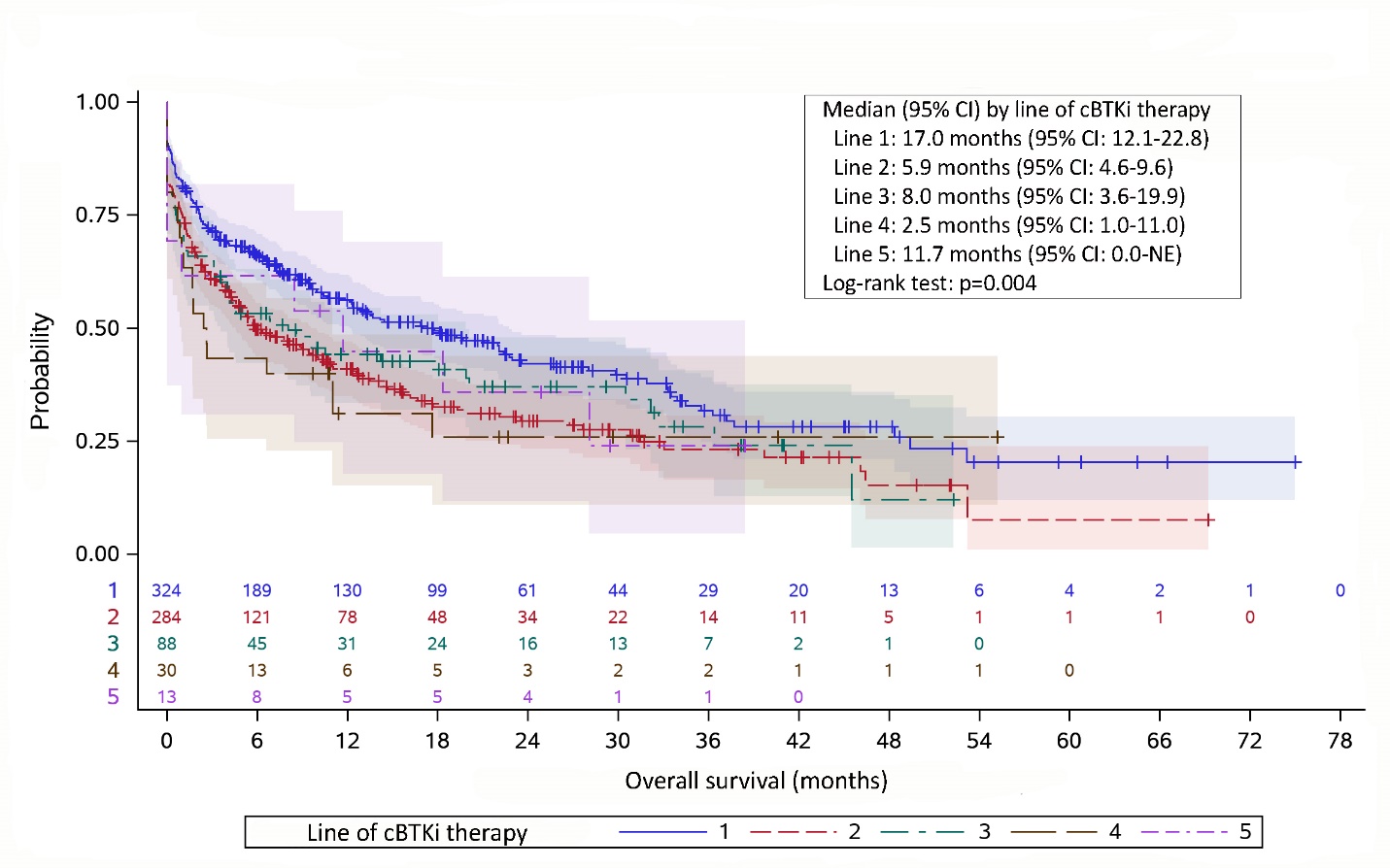
**

Abbreviations: cBTKi= covalent Bruton Tyrosine Kinase inhibitor; CI=confidence interval; NE=not evaluable
